# Dealing with low access to harm reduction: a qualitative study of the strategies and risk environments of people who use drugs in a small Swedish city

**DOI:** 10.1186/s12954-022-00602-y

**Published:** 2022-03-04

**Authors:** Julie Holeksa

**Affiliations:** Department of Social Work, Faculty of Health and Society, Malmö University, Citadellsvägen 7, 211 18 Malmö, Sweden

**Keywords:** People who use drugs, Harm reduction, Needle exchange, Housing first

## Abstract

**Background:**

The development of harm reduction has been limited in many areas of Sweden. This study aims to understand the implications that this has for the life circumstances and risk management of people who use drugs in areas of low access.

**Methods:**

Eleven qualitative, semi-structured interviews were undertaken with people who use drugs in a small urban centre with no needle and syringe exchange program (NSP) or Housing First policy.

**Results:**

Participants reported many solutions to lack of NSP, including travel to an external NSP, creating bridging distribution networks, stealing, borrowing, reusing, ordering online, and smuggling injection equipment. They were at risk of having their equipment confiscated by police. Participants were mostly homeless, and to address exclusion from housing services, were forced to frequently find new temporary solutions, sheltering themselves in public places, with friends, in cars, among others. Participants felt the lack of services reflected stigmatized notions of drug use and heightened their exclusion from general society. For example, they avoided accessing other health care services for fear of discrimination. These issues caused high levels of stress and anxiety, in addition to serious risk for many somatic and psychological health conditions, including HIV and HCV transmission.

**Conclusion:**

Lack of harm reduction services placed a great burden on study participants to develop strategies due to gaps in official programming. It also contributes to a vicious cycle of exclusion from services. The implementation of such evidence-based programs will reduce this burden, as well as provide the indirect, symbolic effect of inclusion.

## Background

The use of illicit drugs is associated with an increased risk of a number of intersecting health [1, 2], psychological [3, 4], and social vulnerabilities [5, 6]. This is especially true of injection drug use, and for those who have substance use disorders [1, 7]. There is a good evidence basis for programs and policies based on the paradigm of harm reduction, such as needle and syringe exchange programs (NSP), opioid substitution therapy, supervised consumption rooms, among others, in mitigating some of these concerns [8–11]. Various forms of well-developed harm reduction programing demonstrate effectiveness in reducing risk behaviours [8, 9], blood-borne virus transmission and bacterial infections [8, 10, 11], serving as the basis for other health interventions [12], improving referral to other health and social services [13–16], and quality of life [17]. Furthermore, they may promote positive engagement with the healthcare system more generally, and qualitative studies on people who use drugs experience of these services demonstrate that they view such interventions as a “safe haven” or “a little heaven in hell" [13, 18–21]. However, how or if these programs are developed and implemented varies greatly both across and within countries.

Sweden has been widely noted to have a restrictive, deterrence-based drug policy, influenced by a “zero tolerance” approach, and an aim to have a “drug-free society”, stemming from the early 1980s [22]. Policy responses are built on this notion, and drug use in Sweden is criminalized and highly stigmatized, with a law enforcement focus on street-level drug use and a history of low access to harm reduction. The zero-tolerance approach has been criticized, due to its ineffectualness and negative impact on the lives of people who use drugs [22–24]. Notably, Sweden has consistently been at the top end of European drug-related deaths reports [25] (though these can be faulted for lack of common methodology). In recent years, there has been a general shift towards increased focus on harm reduction; however, these changes have not been uniform across the country.

Sweden operates a universal healthcare system, which is mostly free at the point of access.^1^ Health and social care offerings are decided at the regional and municipal level, respectively, leading to local service variations. Harm reduction offers a prime example of this variation. While the first NSP were opened in two neighbouring cities in the Skåne region in Southern Sweden already in 1986 (Lund) and 1987 (Malmö), debate surrounding these programs limited their continued development [26–28]. In the Swedish context, NSP have been controversial because it is seen to directly contravene the goal of a “drug free society”. Outside of this region, the first NSP was opened in 2012. In 2017 a policy change led to more widespread development than before, and now NSP operate in 17 of 21 regions. The development of harm reduction, and NSP specifically, in Sweden are well described in Eriksson and Edman [27] and Karlsson et al.[28].

Where offered, the practice of NSP in Sweden, has a specific, rigid characterization, as described by Alanko-Blomé et al. [29]. NSP are located within the healthcare system, often at a hospital [29]. This limits the number of physical locations. They have high entry requirements, including identifying oneself, a minimum age of 18 (previously 20) years, and taking part in mandatory HCV and HIV testing [29]. They are only offered during business hours [12]. NSP offer a full staff of an infectious disease specialty physician, nurses, and social workers [29]. The positive aspects of this structure are access to a wide range of cohesive services and efforts, including social work, sexual health, basic medical care, help to enter drug treatment, among others. Consequences of this programmatic structure include excluding people from care due to time or geographic limitations, or their desire to be anonymous, especially in the previously described context of drug use criminalization. Consequently, the provision of such a program does not mean access in practice. Official NSP are the only place in which sterile injection equipment can be legally obtained by people who use drugs. In contrast, in many other nations, particularly in Europe, needles and syringes are often offered freely or by purchase through, for example, pharmacies, vending machines, and/or NGOs, among others, in conjunction with NSP locations, with no requirement of participation in other aspects, or identifying oneself [30].

The recent improvements in the breadth of NSP coverage also reflect other recent positive changes in the Swedish harm reduction landscape. However, these changes vary substantially on a regional and municipal level. For example, the Skåne region, recently improved access to opioid substitution therapy, which has, until recently, been characterized by a high-threshold model. This involved long waiting lists, with strict criteria regarding an extensive documented history of substance use, regular urine drug testing with expulsion or suspension upon a positive test, high age limits, and limited personal agency/preference of the service users. Some regions have undergone reforms in recent years, which have led to increases in access and patient numbers [31], as well as increase in patients’ sense of empowerment over their own care [32].

Broader harm reduction programming, such as Housing First policies, is also unevenly implemented in Sweden. The predominant model to address homelessness in Sweden can be referred to as a “staircase” or “treatment first” model [33]. This model requires individuals to have stopped all drug use and undergo monitoring to ensure that they can maintain an apartment “adequately” [33]. Conversely, Housing First, as the name implies, offers a stable home as a primary support, where abstinence from substance use is not a requirement to receive or maintain housing, but with the belief that any attempt to address such concerns would be better done concurrently, rather than in a successive manner [33]. The focus is on the stability that a permanent home can give someone as a basis for other interventions, and harm reduction is considered to be a key feature of such programs [33]. Housing First programs were first introduced in Sweden in 2010, beginning in the capital area of Stockholm, as well as in the municipality of Helsingborg, which is in the Skåne region previously noted for its early implementation of harm reduction efforts. The development, interpretation, and implementation of the first Swedish Housing First programs are reported upon in detail by Knutagård & Kristiansen [33]. Evaluations of such projects in the Swedish context have demonstrated positive results, such as improved quality of life and social relations, as well as reduced or terminated substance use [34, 35].

Housing First has been promoted by the Swedish Association of Municipalities and Regions (Sveriges Kommuner och Regioner) [36] and is in the National Board of Health and Welfare’s (Socialstyrelsen) national guidelines since 2017 [37] but has only been taken up by a limited number of municipalities. As of 2020, approximately 40 of Sweden’s 290 municipalities had validated such a policy [38], although it is unknown how many of those actually offer Housing First accommodation in practice. In those municipalities which offer it, Housing First is not an exclusive policy in many cases, and may be offered in parallel with other models [33].

Additionally, take-home naloxone (THN) has recently been made available, but has restrictive access criteria. Since 2017, it is only available to individuals who are themselves deemed to be at risk of an opioid overdose, by prescription [39]. Friends, family members, or individuals who use non-opioid substances cannot be given THN, though they may attend training with the view to administer THN to someone in their network [39]. The legislature states that “the patient [needs to] make sure that the medicine is available to the person who may need to give it” (translated by author) (p. 16) [39]. THN is often distributed through, for example, opioid substitution programs and NSP. There are no supervised consumption rooms or heroin assisted treatment programs currently in Sweden.

### The current case: operationalization of “low” provision of harm reduction

While Sweden continues to move in the direction of harm reduction, some regions and municipalities continue to lag far behind due to the influence of the zero-tolerance approach. There are a number of different ways to operationalize what “low” harm reduction may be. In this case, I have chosen the lack of two programs—NSP, and a Housing First policy—as the primary criteria. These can be seen to encapsulate both a “direct” and “indirect” harm reduction approach.

Despite recommendation by the Swedish Public Health Agency (Folkhälsomyndigheten) [40] and National Board of Health and Welfare (Socialstyrelsen) [41], at the time of study, four regions remain completely unserved by a NSP. The current study takes place in a city in one of these four regions. The city and region of study remain unnamed out of concern for the anonymity of participants in a setting of criminalization of illicit substance use. One hypothetical defence that could be used to not open a NSP in the Swedish context would be lack of population density, relating to the above-described high administrative demands of the program. However, NSP are open in several cities in Sweden with significantly smaller populations than the current case. Therefore, this setting is particularly interesting due to the fact that it is primarily municipal political actors who have constrained this development.

Additionally, while some of the municipalities in this region do have a Housing First policy, the particular city in focus lacks such a program, meaning that individuals who are currently using drugs are excluded from accessing government housing programs. Knutagård and Kristiansen [33] have suggested that the limited take-up of Housing First in Swedish settings could in part relate to the fact that “the core element of harm reduction within the Housing First philosophy challenges the traditional substance abuse work in Sweden, which is based on the requirements of abstinence and control” (p. 102).

Other well-evidenced interventions, such as opioid-substitution therapy, are provided in a limited fashion, in the region, with very few clinics and no choice or agency over care provider from clients. Despite that Sweden in fact was one of the first countries in the world to provide methadone maintenance therapy (1966), proliferation across Sweden was slow, and this region did not begin such provision until several decades later [42]. As stated above, take-home naloxone is often distributed by NSP. The lack of NSP in this region means access is limited, especially for individuals who are not engaged in addiction care, or who do not access other health care services. Together, these factors can be used to demonstrate this region’s reluctance to embrace evidence-based harm reduction practices, leading to low access for the population.

Place is an important determinant of health [43–45], and research has demonstrated the influence of geographic proximity and utilization of harm reduction services and/or engagement in risk behaviours. Studies which have mapped the willingness of people who use drugs to travel for services have demonstrated a direct relationship between distance to and use of services [46–48], syringe sharing [49, 50] and thus associated health outcomes. Harm reduction-based strategies have developed and expanded in many countries in the past few decades. However, as can be seen in a case study by Clarke [51], due to the moralized nature of drug use, some of the programs and services associated with it are seen as controversial. As stated, this means that there are significant variations of service offering and provision both between and also within countries, which mediate access on an individual level.

Studies on regional-level variations or deficiencies in harm reduction provision have taken place in settings such as Canada and the United States. These are often characterized by the urban/rural divide and low population density as the primary factors behind lack of service provision [47, 52–56]. Another group of studies demonstrate that people who use drugs will travel or migrate, in part to access better services [57–61]. The existing studies have also primarily focused on service needs, barriers to care, and potential solutions [47, 52–54, 56]. Fewer studies have focused on urban areas lacking services [52] or about how people who use drugs who choose to stay in areas of limited service-provision experience and cope with this, and the broader implications this has for their social vulnerability and health risks.

The purpose of this study is to investigate the life situation, risk exposure, and risk management for people who use drugs who live in an urban area of a region with low access to harm reduction*.* It asks the question: what does it mean to people who use drugs to have low access to harm reduction measures, in terms of their lives, health, risks, and social exclusion, and what strategies do they employ to contend with this?

## Methods

### Study design and recruitment

A qualitative study was carried out, consisting of semi-structured interviews. The primary recruitment target for the current study was people with an extended history of substance use, with a focus on people who inject drugs. However, one individual with a history of non-injection substance use was also included, due to their history of engagement in harm reduction programs. Recruitment took place in a small but urban area in a specified region of “low” harm reduction service coverage, as described above.

Interviews were conducted over the phone or on Microsoft Teams, between January and March 2021. Due to COVID-19-related complications, a convenience sampling method was adopted. I was unable to travel to recruit for this study in person. Instead, I came into contact with individuals (henceforth referred to as “recruitment partners”) who worked at two organizations locally whose services were primarily aimed at people who use drugs. One organization provided overnight shelter and meals, and the other provided treatment for substance use. I provided them an information sheet digitally, and these recruitment partners assisted me in advertising and recruiting for this study. At one service, I was given the phone numbers of individuals who indicated their interest in participating, with whom I conducted a phone interview at their convenience. At the other service, the recruitment partners set up video calls in their office. The limitations of this recruitment strategy are addressed in a later section.

### Data collection

The interview guide was designed to explore participants’ accounts, key topics included: perceptions of Swedish and local drug policy and its impact on them as individuals, injecting equipment access and use, risk behaviours, experiences of accessing other harm reduction and health services, how lack of access to certain services is dealt with, as well as perceived health and social care needs. Structured questions, with relation to sample characteristics and life circumstances, such as housing and income, and information on history of drug use, were also asked.

In total, 11 semi-structured interviews were conducted, ranging from 21 to 73 min in length (mean duration 49 min). Interviews were recorded with the permission of participants. Participants chose which language (English or Swedish) they preferred to be interviewed in. Interviews were transcribed verbatim. Completed transcripts were proofread and checked for accuracy. Data coding took place in the original language of the interview, and I translated excerpts used for display in the current report. These excerpts were checked for accuracy by two bilingually fluent individuals.

### Analysis

Thematic analysis, as described by Braun & Clarke [67], was chosen as the method to guide the analysis of the interview data, due to its flexibility and theoretical malleability. Qualitative data analysis software (NVivo 12) was used to assist in this process. As interviews took place, I kept memos of initial impressions. Interview transcripts were read and re-read in order to familiarize, and preliminary notes and interpretations were made. I employed an abductive coding method, where some of the codes came from preconceptions and backing in the literature, and others came from the data themselves. Coding was an open, fluid, and flexible process, in which ideas viewed to be relevant or of interest were noted. Codes which were decided upon throughout the initial reading process were then developed and refined through a process of continual revision. Codes (and the data excerpts they represented) which were interpreted to have shared meanings were grouped together to create initial themes. Themes were then checked for consistency and representativeness, and revised as necessary. The aim of developing themes was not merely to summarize the data, but to interpret in order to find core, central, unifying concepts [67]. This allows for the deeper meaning of the data to be clarified. Data extracts were found and chosen to elucidate analytic claims.

### The risk environment as a theoretical framework

Several authors have critiqued individual behavioural orientations to reducing drug-related harm [62–65]. Many harm-reducing programs require that individuals actively seek out care and reduce their own risk, but in reality are limited by “the material constraints on individual human agency” [65] (p. 3036). Rhodes [63, 64] suggests a more contextual, “risk environment” approach to understanding the engagement in ongoing risk or health-seeking behaviours [59, 60]. This approach investigates the systems surrounding the individual, and acknowledges the multiple competing priorities that play a role in risk management [64]. Understandings of drug harms must be seen to be mediated by the interplay of social, policy, economic, and physical dimensional factors [66]. The current study employs these dimensions of the risk environment to assist in interpreting themes and organizing them within the discussion. These may be further examined at the micro- (individual, interaction), meso- (group norms, community level access to services), and macro- (national level policies and laws) levels [66]. Participants’ experiences are situated within their contexts, and where applicable, the role of their environments in producing or reducing risks is examined.

### Ethics

The project on which the current study is based was approved by the Swedish Ethical Review Authority (Dnr 2019-06509). All participants gave their informed consent to participate. All study procedures complied with relevant laws and institutional ethical guidelines. Given the nature of the topics involved, extreme caution was taken regarding exact names, locations, and other identifying personal details when both interviewing and transcribing. Any such specifying personal information was removed from the final transcribed product, in order to ensure confidentiality. Participants were remunerated with a 200SEK gift card for their time.

## Results

### Participant characteristics

Eleven individuals took part in this study, of which ten were male and one was female. The age range was 31–55 years, with an average of 43 years. All but one had injected drugs. Most (eight of 11) had ongoing daily or near-daily injection use. The remaining three had a history of substance use and were linked to the recruiting organizations, as above, treatment for their substance use or a shelter and meal program, and thus were knowledgeable about harm reduction interventions. Ten of 11 reported at least a decade of substance use, and eight reported use beginning in teenage years, or earlier. The primary drug of choice was amphetamines, followed by heroin and other opioids, and benzodiazepines. Alcohol and cannabis use were also reported. Four of 11 reported current polysubstance use. Seven were homeless or unstably housed. One participant was regularly employed, the rest reported a mixture of income generation from state pension, income assistance and/or disability payments, casual employment, begging or busking, and criminal activities.

### Findings

In this section, I present the two overarching, interrelated themes developed through the process of analysis: how individuals in the study addressed their needs in the face of lack of access to harm reduction services, and the health, psychological, and social consequences of this lack of access. These themes are first presented on the direct level, reporting NSP and Housing First individually, followed by presentation of the broader-level consequences and implications of the absence of services.

### Strategies and consequences related to the lack of NSP

The participant population as a whole was made up of individuals with a very high level of consistent need of sterile injection equipment—as noted, most were injecting daily or near daily—which was not being met by formal solutions in their immediate physical environment. They reported that their current city was their home, where their families and social networks were, where they were connected to other services, so, for most, permanent movement to a city with better service access was not an ideal option. They reported a number of different ways in which they got around the lack of access to care in their region. Almost all noted that they had at some point travelled to another region of the country, approximately 100 km away in each direction, to collect a large number of syringes and needles at a formal NSP:

“And then we gather [the used injection equipment] all together. I think I left a hundred syringes and needles that I had at home in a box…And those at the syringe exchange program, they know what situation we’re in up here. So they have been nice… always provided extra tools as well. And I think that is- it makes me really happy”. (Participant 8)

“Yes…it’s far. I live in [town], yes but, it’s a long way there. So it takes a while. But it’s going well, they have nice staff. You are treated well, you’re just anyone. They aren’t like ‘Oh, an addict!’” (Participant 7)

Despite the effort to travel there (some participants noted doing so on a weekly basis), several participants highlighted the additional, psychosocial impacts of such services, further to receiving sterile injection equipment. For example, when discussing the external NSP and the staff there, another participant remarked:

“How soft and welcoming they are and what service they stand for … it’s beautiful.” (Participant 6)

However, not everyone was able to consistently visit an external NSP. Instead, they reported gaining access to equipment “in lots of different ways. From friends, maybe in a burglary… at the veterinary clinic and such. I can order them online” (Participant 4). Among other strategies reported by participants, one reported accessing the hospital ER in hopes that a member of staff would give them spare syringes, one reported fashioning injection equipment out of household items, and a number reported smuggling them from other countries where they are more easily accessible, such as Norway. Another commonly stated solution was secondary syringe and needle distribution, that is, working together amongst a small network to distribute legally obtained syringes to each other. Participants reported both giving and receiving this equipment, either for free or in exchange for drugs, the below accounts demonstrating the concern these individuals have for access in their wider network:

“I have many of my friends, or yes, acquaintances…they do not think because they are addicted. They don’t care if they’ve used a needle for three or four weeks then…. I just – ‘What the hell, why didn’t you call me? You know I’m sitting on my tools. How hard is it to call me and ask me, do you have any tools? Because you know I’ll say yes. … Yes, come and pick me up then. Replace your old ones that you have, so I can take them. So you can take new ones from me instead.” (Participant 6)

“There are many who do not dare to go there [the external NSP], or who cannot. And then I think they have as much right as I do, so then I go there and pick them up for us… I get it for free, they get it for free … You should not have to pay to get well, or stay healthy.” (Participant 7)

This sort of community cooperation was key in maintaining their health and wellbeing, and was found both for syringe distribution, as well as finding places to sleep and inject together. In this case, their social environments functioned as a manner to mitigate risks at the micro-level. Many noted the importance to them of ensuring that people in their network were using sterile equipment, as “we addicts have a special belonging to each other. We actually take extra care of each other a lot” (Participant 6). However, while this may reduce the hindrance of travel and time for some, it may increase the burden for others, placing them into a sort of informal, uncompensated provider role. It also may lead to inconsistency in equipment access.

Despite their efforts, participants faced a number of challenges to consistently accessing new, sterile injection equipment. For those who did report going over distances to access an external NSP, personal compromises were made, for example, noting:

“[If there was a NSP in my city], I wouldn’t have to go so damn far, spend a whole day in another city, which I don’t even like… And then yes, more children, grandchildren I could spend my time with. So it’s a lot. So I would get a lot of time for other things.” (Participant 7)

The hidden costs of health risk management included travel time, money for transportation, general discomfort in a different setting, and lost time with loved ones. Compromises were being made between accessing the NSP and other important priorities, including important social connections with family.

Unsurprisingly, a primary consequence of lack of NSP and other low threshold services was the extensive reuse of injection equipment, sharing with others, and the acquisition of used injection equipment. One participant explained:

“I have to use the same syringes for like, well, at least 5 days … like 15 times, it’s no problem … Some people are going on travels to [town with NSP]. But I’m not in that capable to go on busses or trains or like that.” (Participant 3)

Another participant’s comments illustrated the issues with insecure access and relying on others for injection equipment:

(Participant 9) “… And now I order or I get from others.”

(Interviewer) “Yeah so you always get clean ones?”

(Participant 9) “Not always.”

(Interviewer) “… When you get them from other people do you pay for them? Or do you they give them for free?”

(Participant 9) “No [I don’t] pay for them, I think we pay enough to chance if you’re going to get HIV or something else just by using it.”

Thus, relating to the dimension of the physical environment, the geographic impediment to access has clear and important implications on engagement in risk behaviours for this population. This has important impacts on health outcomes – including blood-borne viruses such as HCV, for which Sweden provides free treatment – which then “goes around and around like a hamster wheel” (Participant 3) due to ongoing lack of sterile equipment access leading to repeated HCV reinfection.

### Strategies and consequences related to the lack of stable housing

As noted, this area offered a “treatment first” model of housing and, accordingly, people who use drugs were excluded from housing programs. Participants reported a number of strategies in which they addressed this themselves. Most were homeless or unstably housed, and found more or less temporary housing through shelters or by friends and family. Others reported solutions such as “sleeping in my car, and a storage unit” (Participant 5). For those who had apartments, any ongoing substance use meant that “it hangs by a thread whether I can keep it or not” (Participant 8).

Due to the lack of formal solutions, they reported moving frequently, often faced with short-term solutions, unable to settle in any one place. Those who were actively homeless in particular faced a nightly struggle. One participant’s description of their everyday existence exemplified this effort:

“So then I have been at the train station until half past two… Then they close there, then I was out walking in town then. And I have a pretty heavy pack … just because I never know what’s coming. So I probably have a 50 kg pack, which I go and carry. So it becomes a little hard there to carry… But it will be like carrying on a home as well. But yes, I went from there down to the town, the public toilet, so we stood in a public toilet … And there we still stood until five o’clock this morning, approximately. Because then we knew that by then the train station had opened, they open at a quarter past four… So you could go there again then…until eight, before I came here.” (Participant 6)

Similarly to how they addressed lack of NSP, harnessing community contacts was one strategy to facilitate short- or long-term housing. Here again we can see how micro-level social environments can reduce harms. However, it also may lead to issues for those friends and family who must provide space for them, seen in an example by a participant who was asked to leave housing which was being provided by a friend because “I started with the amphetamine. And then people came in, and he [the friend providing housing] did not really want any people there running around, he wanted peace and quiet. And it ended up being a lot of running around. And so, I lost that one too” (Participant 5). This illustrates the tension between wanting to assist a friend, and the issues with trying to deal with some of the lifestyle factors surrounding substance use.

The further stress that this instability and patchwork of informal strategies causes may exacerbate the health and social issues related to both substance use and homelessness. Housing was seen as one of the foundational elements of wellbeing because “you need a home, you need a safety” (Participant 9), but in this case “the help network, it starts in the wrong end” (Participant 9), referring to the fact that cessation of substance use was required in order to get housing, when in fact the participant felt that housing would help support the achievement of abstinence. The needs of the local authorities, rather than the needs of the participants themselves, dictated what services they received.

Lack of housing options for people who use drugs is a particular problem in cities such as the one in focus, where average winter temperatures may be well below freezing, and is highly problematic for the participant population. This, in addition to persistent housing insecurity, gave participants a high level of stress about their futures and in some cases contributed directly to their ongoing substance use as a way to cope. This strategy is one which then may cause a vicious cycle of exclusion from housing services. As one individual described this dilemma:

“If…I’m homeless, well then I have to find a way to survive, and that’s with the drugs. … That’s the only way I know to survive in the rough I mean. You have to take something to, it’s like 11 degrees below zero now, so it’s cold here, so the only way to keep awake is by using something.” (Participant 3)

Those who were homeless and using substances were excluded from municipal housing, and some of those who were housed noted avoiding injecting at home over concerns of eviction. Thus, lack of low-threshold housing programs free of abstinence requirements (as well as lack of a supervised injection facility) led to public injecting “usually in the toilet. Often in the toilet so you can get water. Sometimes with Coca-Cola, or something like that. Once out in the woods, I took water from the ground. Yes, there have been some shady situations at times. But that’s how it’s been” (Participant 8). This was reported by almost all participants, particularly occurring in public toilet facilities. One participant noted that they were potentially going to lose their housing due to their substance use, and described public injection as a way around this, but coming with its own risks, stating:

“I use in public toilets sometimes. Well of course I’m getting vulnerable, if I’m using in a public toilet abusing and then going out from the toilet and the cops are coming or something. Then you get problems.” (Participant 2)

In this case, the participants had to weigh the options of potential arrest versus the possibility of losing their home. This has implications for overdose and bacterial infections in particular, as well as consequences for social issues, such as public persecution, arrest, and stigma.

### Broader consequences for health and social exclusion

Beyond the direct concerns reported by participants, there were several notable references to the indirect impacts of the lack of these services on their health and wellbeing. These largely stemmed from the macro, policy-environment, and how this affects attitudes towards and inclusion of people who use drugs. The development of both Housing First and NSP in the current municipality is primarily a political question, as the National Board of Health and Welfare and/or the Public Health Agency have promoted their development, while municipal actors have delayed their implementation. Seizure of (legally or illegally obtained) injection equipment by law enforcement was commonly reported. In regions with NSP services, staff work with and educate law enforcement on the program, to limit such practices. In this region, this cooperation does not exist and participants had limited recourse to address these confiscations, citing “what shall I do? I go to the needle exchange again” (Participant 1). The presence of harm reduction in the policy environment may lead to cooperation and bargaining between different actors (particularly official actors who have legitimacy over such concerns in the eyes of law enforcement), improved understanding of such issues, and consequently better relations between all groups.

In contrast to the above-reported high quality and non-judgmental services accessed at the NSP externally, participants “have as little to do with healthcare as possible” (Participant 6) in their own region, because of lack of trust, or a poor therapeutic relationship. Participants felt that the lack of low threshold harm reduction programs was reflective of other, stigmatizing features of healthcare locally:

“Doctors usually think that I am a drug addict who lives dangerously.” (Participant 4)

More broadly, participants were keenly aware of their deviant position, and reflected that general society made them feel that “if you are homeless, a drug addict, then you are dirt” (Participant 7). Mirroring that sentiment, another individual discussed how they resisted that identity when possible:

“I wash and maintain my hygiene, and so on. I know that it is difficult for many who end up on the streets. I want to keep my hygiene, so that no one can think ‘Oh but [they], [they abuse]. You see, [they smell] disgusting.’ Or yes, I do not want to be that kind of person. I want to be this person, I have an image that I have to maintain.” (Participant 6)

They also felt that the possibility to access certain services would signal a macro-level inclusion and acceptance in society in a different and hopeful way, in the words of one participant:

“The people are thrown in the wind. They are sleeping in the train station, people are outcasts. They are not feeling a part of the society. Involved in the society. [Such services would] inform them, ‘this is what works, this is what we can afford you’. Then they are getting closer to the society and then they are maybe someday they are thinking again.” (Participant 3)

Ultimately, this level of exclusion, the multiple competing priorities which it causes, and overall lack of knowledge of what might happen in both the immediate and distant future. Several of the individuals who were interviewed reflected that this was a constant stress and negative influence on their mental wellbeing:

“I may not be feeling really well up here [points to head] … You think a lot, what will my life look like? How will it end? Where should I get money from? How do I solve certain things? And-, yes, where will I sleep? …It’s so much, like, and that’s something that takes a lot of energy.” (Participant 5)

“You get very lonesome. You can’t go talk to anyone, and also because of the policy in Sweden, it’s very hard. If you go try to seek help you self-incriminate yourself. … It was very nerve wracking to go seek [care] because I had kids at that time. So … I knew [it was possible] that Social Services would take contact with me and, like, investigate me as a parent.” (Participant 10)

The above quote demonstrates the impact of both real and perceived potential discrimination. The punitive consequences of their drug use lead to reticence to access such services which are offered, such as opioid substitution therapy and other substance use treatment programs, or social services. The highly stigmatized and criminalized nature of drug use in Sweden means that the benefits of service access do not always outweigh the potential consequences of identifying oneself as a person who uses drugs. Another participant reflected on the relief they would feel to not be constantly chased:

“[if drugs were decriminalized for personal use] maybe [I’m] gonna take a deep breath, exhale.”. (Participant 9)

In addition to the measurable outcomes of harm reduction programs, these comments speak to the non-quantifiable impacts of such policies, how they improve interaction with official authorities such as law enforcement, and improve individuals’ psychosocial wellbeing and sense of belonging in society.

## Discussion

### Physical environment

Engagement in risk-taking behaviours exists at a nexus between the individual and their broader environment. It is clear that the lack of NSP, as well as a Housing First policy, in the locality of study has a greatly negative impact on the individuals in the study, the risks they take, and the ways in which they address those risks. The onus of the procurement of sterile equipment has been entirely placed on the individual who is injecting. The availability (or lack thereof) of syringes is one of the primary factors leading to syringe sharing [68–71]. Most individuals in the current study reported sharing injection equipment at least at some point, which has important health impacts, such as leading to the avoidable transmission of HCV, other blood-borne viruses, and bacterial infections [1, 2]. By offering easily accessible sterile equipment, this risk behaviour can be significantly reduced. Public injection, too, is highly associated with negative outcomes [72]. Such situations often compel individuals to inject in a rushed manner, in an unsterile setting, with no professional overview in the event of an overdose [73]. In the current study, the threat of eviction due to substance use meant that even some of those who were housed often injected elsewhere from their home, to avoid detection.

Despite a number of barriers in their way to do so, most participants in the current study engaged in active and committed health-seeking behaviours, and tried to reduce the above-stated risks which are being produced or amplified by lack of services in their own community. Participants described numerous important trade-offs that they made in order to consistently use sterile injection equipment. However, these barriers could prove to be too great at any given time, leading to an increase in risk taking. Contrasting from other studies [47, 54, 56, 74], where programs such as mobile, postal, or pharmacy distribution of syringes have been implemented, official solutions to bridge the gap have been limited in this setting, due to legislative limitations. Instead, informal solutions prevail, such as secondary distribution systems. A significant body of literature on both formal and informal secondary, bridging injection equipment distribution systems exists [47, 75–85]. Such arrangements make up a large proportion of needle and syringe distribution in some settings [75, 80], and may allow for more geographically widespread distribution, as well as the engagement of individuals who do not or cannot engage in more formal programs, such as younger people [80, 81, 85]. However, these solutions are not always sustainable or consistent[79], may be limited to social networks [83, 85], and may not reduce risk behaviours as effectively as formal sites [82, 83]. The data from this paper confirm the importance of such practices. However, the data also demonstrate the potential burden this has on those who are seen as “providers”, as well as the imperfect nature of the solution, as recipients have to pay for or rely on the generosity of others for equipment access. Therefore, informal secondary exchange should not be relied on to make up for official service deficits in a long-term manner.

As previously described, longer physical distances from services lead to reduced access and increased risk behaviours [46–50]. As one strategy to address distance-related issues, other studies have highlighted a migratory element with relation to accessing services, with focus on people who use drugs [58, 59, 61, 86–90], as well as the interlinked field of mental health and homelessness services [91–94]. Perez Torruella [59] describes that such migration is encouraged by Puerto Rican authorities, on the basis of improved treatment access. However, there are also risks associated with this. Other studies have found that recent migrants in a period of “transition” were significantly less likely to access harm reduction and social support services, or be more likely to engage in risk behaviours [86, 88, 90, 95]. The individuals in the current study noted having important connections locally to family, children, social networks, social services, among others. Therefore, permanent migration may contribute to their further marginalization in a new location, where they would be lacking social connections and knowledge of the harm reduction landscape, and should not necessarily be encouraged. That being said, the extensive, frequent travel which the participants reported embarking upon also must be solved.

From a micro- (individual, interaction-level) and meso- (community level, access to services) perspective, the physical risk environment in this setting produces a significant amount of risk and harm. These levels were highly intertwined and interacted with each other, for example, that actions at the level of the individual were often found to make up for absence of services on the community-level. However, this creates complications of its own, as seen with the reliance on social networks for secondary distribution.

### Social environment

Not only are the direct risks of the physical environment apparent, the setting of limited harm reduction reflects broader social aspects of these individuals’ lives, which produce, or in some cases mitigate, harms. Goffman [96] describes a process by which certain socially stigmatized attributes or identities, such as that of a person who uses drugs, are ascribed to individuals, creating a “spoiled” or “discredited” identity. It has been suggested that stigma functions on many levels as a social determinant of health, exacerbating processes related to stress, isolation, and reduced access to resources [97]. Phelan et al. [98] posit that stigma against groups such as people who use drugs functions, in part, to enforce social norms. Lack of social acceptance towards drug use on the macro-level is acknowledged as an important part of Swedish drug policy, for precisely this purpose [99]. However, this has a twofold effect of internalization of stigma, as well as the experience of discrimination, and leads individuals with this group’s identity ascribed to them being excluded from society. Some participants in the current study made explicit reference to how they managed and resisted being ascribed this “discredited” identity and the stigma that is attached to it. Research on the importance of such identities—both societally and self-attributed—has shown how they may lead to exclusion from the mainstream and/or retention in a drug-using “subculture” [100–104].

Correspondingly, Bourgois [62] discusses the notion of the “moral economy of sharing” representing a “political economy of survival in fragile networks and marginal communities” (p. 2332), with relation to sharing of drugs and equipment in an effort to utilize micro-level social relations to construct supportive networks, resist arrest, conserve resources, and economize drugs. These statements have been further supported by subsequent studies on secondary equipment distribution [76, 84, 85]. A moral economy can be seen in this study, with the protective and collective nature of these communities. Individuals here utilized and harnessed their social networks as a system to receive and distribute materials and reduce their risks. However, this subcultural reliance also risks what Coumans and Knibbe [104] call “hardening”—an adaptive process of behavioural change and coping strategies, which may reduce ability to fit in with the norms of “general society” in the long run. Programs such as Housing First work to combat this, as they may promote social integration, through their incorporation into standard housing complexes. Reports from the Housing First program in Helsingborg demonstrated that tenants had broadened and diversified their social networks, as well as improved relations with their children or other family members [34].

Stigma against drug use may also result in individuals feeling unworthy of care [105]. This was visible in the self-stigmatizing discourse that these individuals have internalized and use about themselves. Internalized stigma is associated with lower utilization of NSP [106], and negative mental health outcomes [107]. Additionally, real or perceived trusting or discriminatory relationships with healthcare providers also is a factor which mediates outcomes amongst this population, particularly healthcare utilization [108–110]. The impact of these micro-level interactions, as well as macro-level policy context, is reflected in the participants’ reported lack of desire to attend medical services in the region, due to the perceived discrimination they face. Low-threshold services may provide a conduit through which to engage and generate trust, even in the broader context of stigma in society. These programs not only provide a specific service, but also may be a first point of engagement, to “coax NSP clients (quite literally) to a place where they can express, often very strongly, their trust in the service”. [21] (p. 143). Stenström [111] found that many individuals engaged in the NSP in Malmö often participated in “social” visits, without explicitly engaging in services, and that such contacts were a predictor of lower HIV/HCV incidence. This was hypothesized to relate to increasing the integration of clients in care and general society. The development of a trusting therapeutic relationship with social workers has also been reported amongst those in a Swedish Housing First program [34]. This may also lead to a renewed self-esteem amongst participants.

### Policy environment

In this setting, the macro-level “public and legal context of risk management” [63] (p. 89), here being the criminalized legal status of drugs and personal drug use, leads to high levels of social marginalization. Though, certain macroeconomic and societal welfare policies may mitigate some level of harm, for example, almost all individuals in the study were benefiting from some form of government-based income assistance programs. Sweden has a wide and relatively well functioning social safety net and operates largely on a social democratic welfare model, which implies high levels of supports [112]. However, the policy context of criminalization justifies the exclusion of people who use drugs from services. Given the primary problematization of people who use drugs as criminals, their agency and ability to steer their own care is limited in this context. Instead, authorities exert punitive control over them by limiting access to certain forms of care. Moralized assessments of “deservingness” influence who has access to such programs, as well as which programs are offered [41, 49, 113].

With relation to exclusion from housing, homelessness and drug use are often described as a situation of “double jeopardy” which serve to reinforce and compound each other [114]. Lack of housing leads to increased risk of HCV and HIV [115], public injection [116], as well as increased drug use for coping with emotional or physical pain [114, 117], all of which were directly reflected in the participants’ narratives. Housing First policies have demonstrated success in providing housing stability to vulnerable populations [33, 34, 118–120]. Padgett [121] describes housing as providing security in the sense of physical shelter, as well as “ontological security, the feeling of well-being that arises from a sense of constancy in one's social and material environment which, in turn, provides a secure platform for identity development and self actualization” (p. 1926). It is clear that the participants in the current study would benefit from both forms of security. Conversely, putting the onus on themselves, friends, and family members to solve their housing issues leads to a grey area of “hidden homelessness”—provisional accommodation without the security of official tenancy [122]. Such situations may be tenuous, straining relationships on both ends, and leaving individuals vulnerable to the goodwill of others, at risk of exploitation, among other negative outcomes [123].

The macro-policy context also influences the possibilities and focus of police interventions, which have important impacts on the study population [124]. Institutional contexts shape policing priorities and culture [124]. Studies in various cities have shown that directed police interference and stops led to decreased accessing of NSP [125, 126], increased syringe sharing [127], and led people who use drugs to carry fewer sterile injection materials on themselves [128]. It has also been found that such policing practices lead to unsafe public injection practices, due to the stress of potentially being arrested [128]. These practices, while technically legal, should be reviewed and discouraged.

As a whole, the macro-level policy of zero-tolerance towards drug use, and the stigma it engenders, trickles down to the meso-level of missing services and exclusionary policies. At a micro-level, it also leads to a reticence among some participants to engage in official services, where they do exist, for fear of discrimination, and/or repercussions for themselves or their families. Participants in the current study described stresses and an overall sense of insecurity and uncertainty about their futures. This was reflected in the large proportion of participants who reported using drugs to cope with their current life circumstances. Per Keane [129], “social stigma itself could qualify as a harm of drug use and since illegality produces some of the most obvious harms of drug use, investigation of drug laws is also well within the scope of harm reduction” (p. 228).

### Limitations

The current study has a number of limitations. Firstly, due to COVID-19, I was unable to recruit participants directly. I initially had planned to travel to the location of study, and recruit both through relevant organizations, as well as on the street, and through snowball sampling. Instead, I had to rely on recruitment partners from two organizations to recruit on my behalf. This has inherent limitations due to lack of knowledge on how systematically these individuals were advertising or recruiting for the study. I attempted to address this by providing a thorough information sheet and background of my study and having frequent contact with these partners. Ultimately I was limited in my recruitment and was only able to connect with 11 individuals. Attempts to recruit more individuals were unsuccessful due to lack of time resources on behalf of the recruitment partners and their organizations. Nonetheless, the interviews generated rich data of interest and relevance to the study question. Inability to recruit directly or expand beyond the organizational setting has limited my participant population. Individuals who were not participating in either of the programs with which I was in contact would have been missed entirely. This may have included both less marginalized and more marginalized individuals, whose experiences may have been different to the individuals who were engaged in these two programs.

People who use drugs may be reticent to participate in interviews, particularly online, due to the criminalized and stigmatized nature of their activities. This may have also influenced responses due to social desirability biases. Some interviews took place in a private office within the organization of recruitment, which may have limited or discouraged participation for some, due to privacy concerns. Furthermore, interviewing a marginalized population involves building a rapport, often through casual contact, which can begin as early as the recruitment stage in making hesitant potential-participants feel comfortable with participating in an interview, knowing that they will not face judgement or stigmatizing attitudes. As I was unable to recruit individuals directly, those who have faced more discrimination in their life and thus fear this may have opted to not participate. This may be seen particularly in that, despite efforts to reach them, only one female was engaged in this study. It is well documented that women who use drugs (especially who are pregnant or have children) in these situations face additional challenges in identifying themselves to services and accessing care [130–132].

Relating to transferability, the small sample size means that attempts to transfer the results to a different population or setting must be done so with caution. Relating to the above limitations, the findings should be especially carefully applied to the lives of women who use drugs. However, the results may be able to inform the experiences of similar groups (people who use drugs) in similar settings (areas without services for this population), and what consequences this has on their somatic health and psychosocial wellbeing. Resistance to harm reduction and variations in such program delivery are not unique to Sweden. Therefore, the current case could elucidate upon the conditions faced by people who use drugs beyond the current context. Especially  relevant might be regional or national border areas, commuter towns, and other such settings where policies and services may vary widely in relatively geographically small areas.

## Conclusion

Despite decades of evidence, the development of key harm reduction programming has been slow in many Swedish municipalities and regions. The results of this study demonstrate the burden that this delayed, uneven development has had on individuals who are affected by such policies. They must resort to travelling for, buying, stealing, re-using, and sharing their injection equipment. Much of this comes at great personal cost—whether it be time away from family, money for travel, or the potential health impacts of sharing or re-using items. It also reflects further marginalization in other policies, leaving many of the participants in a situation of great insecurity in life, particularly when it comes to housing, as most were unstably housed and many of those who had housing felt constantly at risk of losing it. These participants reflected on their excluded position in society as a whole and internalized their stigmatized identities. NSP and Housing First in particular were focused upon, due to their direct, as well as symbolic, value as interventions of inclusion. Lack of harm reduction services has placed a great burden on individuals to develop strategies to address this, leads them to engage in avoidable risk behaviours, and has created a cycle of marginalization of this population.

## Data Availability

The datasets generated during and/or analysed during the current study are not publicly available due to privacy concerns.

## Abbreviations

HCV: Hepatitis C virus
HIV: Human immunodeficiency virus
NSP: Needle and syringe exchange program(s)

## Footnotes

1 Patient fees of 200kr-300kr (20–29 EUR/23–34 USD) for visits with doctors, up to a cap of 1100kr (108 EUR/127 USD) per twelve-month period.
